# Lipid remodelling: Unravelling the response to cold stress in *Arabidopsis* and its extremophile relative *Eutrema salsugineum*

**DOI:** 10.1016/j.plantsci.2017.07.017

**Published:** 2017-10

**Authors:** Cristina Barrero-Sicilia, Susana Silvestre, Richard P Haslam, Louise V Michaelson

**Affiliations:** Plant Sciences, Rothamsted Research, West Common, Harpenden, AL5 2JQ, UK

**Keywords:** Lipidome, Cold-responsive genes, Transcription factors, Abiotic stress, *Eutrema salsugineum*

## Abstract

Environmental constraints limit the geographic distribution of many economically important crops. Cold stress is an important abiotic stress that affects plant growth and development, resulting in loss of vigour and surface lesions. These symptoms are caused by, among other metabolic processes, the altered physical and chemical composition of cell membranes. As a major component of cell membranes lipids have been recognized as having a significant role in cold stress, both as a mechanical defence through leaf surface protection and plasma membrane remodelling, and as signal transduction molecules. We present an overview integrating gene expression and lipidomic data published so far in *Arabidopsis* and its relative the extremophile *Eutrema salsugineum.* This data enables a better understanding of the contribution of the lipidome in determining the ability to tolerate suboptimal temperature conditions. Collectively this information will allow us to identify the key lipids and pathways responsible for resilience, enabling the development of new approaches for crop tolerance to stress.

## Highlights

• Higher plants rapidly remodel cellular lipids in response to environmental perturbation and abiotic stress.
• Lipids in plants perform many important functions including information exchange, protection, energy storage, signalling and light capture.
• Increases in unsaturation are a common response to cold stress, but not the only mechanism of adaptation.
• An increased understanding of lipid turnover is essential to establish the contribution made by the lipidome to plant stress resilience.
• Opportunities exist to improve the resilience of crops by manipulating the lipidome.

## 1 Introduction

Increases in the frequency, severity and duration of temperature extremes are anticipated to be a frequent feature of our weather. Climate change is driving greater temperature fluctuations, resulting in frequent periods of cold temperatures. Susceptible crops exposed to cold temperatures have impaired growth and development, this limits the use of valuable varieties and lowers yields. Therefore, it is important to understand the mechanisms underlying cold stress responses in the search for more tolerant crops and those that maintain yield under temperature stress. Sensing and reacting to cold stress is a complex process that appears to require a multitude of mechanisms and pathways. The strategies that plants utilise to endure stressful conditions are varied and involve a multitude of molecular, metabolic and physiological adaptations. These strategies make changes that occur in the first instance to protect the plant, followed by cold acclimation enhancing the plant survival under the low temperature stress [1], [2]. Although many of these processes are mediated by transcription factors (TFs) that activate stress-related gene expression, the response of plants to cold is not limited to the transcription network [3], [4]. As a major component of plasma and endo-membranes, lipids have a recognizable structural role in mitigating the impact of cold temperatures [5]. Plant lipids as a group exhibit great structural diversity from simple lipids such as free fatty acids to complex lipids such as sphingolipids. This large diversity comprises a wide range of physical and chemical properties enabling their involvement in a wide variety of physiological processes. They constitute a varied group composed of different forms and structures, typically divided into eight main groups, fatty acids, glycerolipids, glycerophospholipids, sphingolipids, sterol lipids, prenol lipids, saccharolipids and polyketides [6]. The unique physical properties of the different lipid species confer a multitude of biological functions involving energy storage and plant metabolism with active surface protection, structural and signalling roles. Biological membranes have a crucial role in the protection, homeostasis, and metabolism of the cell. There phospholipids are combined with sphingolipids, sterols and proteins that interact with, and have active role in metabolism [7]. Indeed, cold temperature resilience is often defined by the ability of the cell membranes to adapt [8], [9], [10].

Extremophile plants, that can endure extremes of abiotic stress, are often proposed as models to study resilience to severe environmental conditions. One such example is *Eutrema salsugineum*, an extremophile belonging to the Brassiceae family, which is highly resistant to salinity stress, (hence it is commonly known as ‘salt cress’). In addition to its halophytic properties it also shows a high tolerance to drought and cold stress. Furthermore, it has a close evolutionary relationship with the model plant *Arabidopsis thaliana* (*Arabidopsis*), with high genome homology and similar plant morphology [11], [12], [13]. These characteristics are extremely advantageous, and thus *Eutrema salsugineum* (Eutrema) is now utilised as a model for abiotic stress studies [14].

This review presents an overview integrating gene expression and lipidomic data published so far in *Arabidopsis* and its relative the extremophile Eutrema, to better understand the contribution of the lipidome in determining the ability to tolerate suboptimal temperature conditions. Collectively this information will allow us to identify the key lipids and pathways responsible for resilience, enabling the development of new approaches for crop tolerance to stress.

### 2 Cold stress: symptoms, sensing and strategy

Plants are poikilothermic and sessile, as such they are highly influenced by the surrounding environment. Of the many abiotic challenges faced by plants, low temperature often defines the natural distribution of plant species, and limits the survival range of many agricultural crops. Cold stress reduces plant growth and development, altering the physical and chemical composition of cell membranes, causes electrolyte leakage, decreased protoplasmic streaming and changes in cell metabolism [2]. Further responses to cold include changes in nucleic acid and protein synthesis, enzyme conformation and affinity, water and nutrient balance and impairment of photosynthesis, specifically the down-regulation and photo-damage of photosystem II (PSII) [15]. Despite the deleterious effects of suboptimal temperatures plants can endure low non-freezing temperatures, which increase resilience to stress and extend the temperature range in a process called cold acclimation [2], [16]. Our understanding of how plants respond to cold temperatures has come through investigations of the regulatory network underlying cold acclimation. The process is complex and system-wide, incorporating turnover at the transcriptomic, proteomic and metabolic levels. [4], [17], [18]. The changes in protein and lipid membrane composition help to restore metabolite homeostasis and are considered a mechanism by which cells sense cold temperatures [9]. The fluid state of the plasma membrane is a structural and functional asset for its metabolic and physical role. The plasma membrane transitions from a fluid state at high temperature to a rigid gel phase when low temperatures are present. Low temperature-induced changes in the physical conformation of membranes are mainly due to the increased level of unsaturated lipids enhancing membrane fluidity and stabilization, allowing cells to mechanically adapt to cold [19], [20]. Sugars, nitrogen compounds and proteins produced during cold acclimation have an active role in stabilizing both membrane lipids and proteins, conserving membrane integrity [17]. Other sensing mechanisms, closely related to lipid turnover, but not fully understood, involve Ca^2+^ channels, kinases and G-protein associated receptors. Cold temperatures typically trigger a signalling cascade which includes Ca^2±^ flux and −dependent protein kinases (CDPKs), mitogen-activated protein (MAP) −kinase mediated cascades, and the consequent generation of lipid signalling molecules e.g. phosphatidic acid, diacylglycerol and inositol phosphate, which collectively contribute to responsive gene expression [17], [21], [22].

The accumulation of abscisic acid (ABA) in response to abiotic stress mediates signal transduction and gene expression. The role of ABA in the cold stress response results from its function as a plant hormone, regulating growth, sugar content, leaf senescence, seed dormancy and germination, cell division and elongation [23], [24]. One contribution of ABA to cold acclimation is its role in promoting phospholipid [inositol 1,4,5-triphosphate (Ins(1,4,5)P3)] metabolism [25] and the generation of second-messengers [21], [26]. However, in the low temperature response pathway, ABA is only transiently accumulated at low levels [2], [24], [27]. Accordingly, changes in plant gene expression in response to chilling are triggered by both ABA-dependent and ABA-independent mechanisms [2], [24]. ABA-dependent gene expression works through an ABA RESPONSIVE ELEMENT (ABRE) in the promoter region of the induced genes, to which ABF proteins (ABRE-BINDING FACTORS) bind and activate expression [24], [28], whereas ABA-independent gene expression is regulated by the CBF/DREB1 (C-REPEAT-BINDING FACTOR/DEHYDRATION RESPONSIVE ELEMENT-BINDING FACTOR 1) TFs regulon, which specifically binds to the CRT (C-REPEAT/DRE) element in the promoter regions of cold induced genes. An ever-increasing number of cold inducible genes known as COR, KIN, LTI, and RD (COLD-REGULATED, COLD-INDUCIBLE, LOW-TEMPERATURE-INDUCED, and RESPONSE TO DESSICATION, respectively) result in regulatory and functional proteins that intervene in the cell metabolism to adapt to low temperatures [29]. As a consequence, profound changes in the cell proteome occur, and these can be distinguished by their structural, regulatory and osmo-protection functions [30].

Low temperature-induced gene expression can be described as transient or long term. Transient expression is generally considered up to five hours of cold exposure, whereas long term expression genes are transcribed and remain activated during the entire cold stress period. Transcriptome analyses in *Arabidopsis* and other economically significant species, such as rice and wheat, have yielded a multitude of potential candidate genes participating in the low temperature stress response; however, some of their functions are still unknown [31], [32], [33]. Extended analyses within the *Arabidopsis* Functional Genomic Network Project, AtGenExpress, has allowed the detection of 24000 protein-encoding genes via the Affymetrix ATH1 gene chip, enabling the identification of more than 2000 cold response genes (1335 up-regulated and 1061 down-regulated genes), of which more than 170 genes encode cold-induced TFs [31]. Although there are several families of stress induced TFs, a significant portion of the cold induced genes are activated by the ABA-independent CBF/DREB1 regulon in *Arabidopsis* [34], [35]. The regulation can be up- or downstream of transcription, and it is activated by low temperatures through a complex network controlling the expression of many genes. Transcriptional regulation during the cold response of plants has been extensively reviewed [15], [16], [29]. Therefore, we will focus exclusively on those that have been demonstrated to modify the cellular lipid profile during a stress response.

### 3 The case for extremophiles: *Eutrema salsugineum*

Knowledge about how plants can cope with extreme environments is economically valuable as these useful traits could developed in crop species. Extremophile plants operate in the most challenging environments, such as extreme cold, drought and salinity, in combination with a broad range of other stresses [36].They are proposed as model plants for stress resistance mechanisms. The model plant *Arabidopsis* has tolerance mechanisms restricted to mild, but not extreme conditions even amongst the most resistant ecotypes [37]. Eutrema (Brassicaceae family) species are extremophile plant models, not only because of their extraordinary resilience to several extreme abiotic conditions, but also due to their close evolutionary relationship with *Arabidopsis* [12], [13], [38]. Based on chloroplastic (ndhF – NADH Dehydrogenase F) and nuclear (PHYA − Phytochrome A) markers [39] the phylogenetic position of Eutrema in the Brassicaceae family was assessed revealing that its separation from *Arabidopsis thaliana* and Arabidopsis *lyrata* occurred approximately 43.2 million years ago (MYA), and from *Schrenkiella parvula* (or *Thellungiella parvula*), a close relative of *Brassica rapa* [40], 38.4 MYA [41]. Commonly known as “salt cress” due to its remarkable resilience to salt stress and characterized as a halophyte [42], [43], it is also exceptionally resistant to cold [12], [38] and drought [44].

Eutrema, formerly known as *Thellungiella salsuginea* [45], has several ecotypes found in China, Russia, Kazakhstan, Canada, and USA [46]. The Shandong ecotype is the most studied and it is originally found in the North Eastern coast of China [13]. Eutrema has a life cycle of 2–2.5 months and is similar in structure and size to *Arabidopsis*, although its leaf shape may change with the growth conditions, appearing waxier and serrated. Despite similarities in flower morphology between *Arabidopsis* and Eutrema these two species cannot be crossed successfully [47]. The genome of Eutrema [41], [43], is approximately twice the size of *Arabidopsis* with 95% homology [48]. The similarity level between genomes of Eutrema and *Arabidopsis* allow a direct comparison between genes and their function, rendering most of the experimental procedures developed for *Arabidopsis* highly adaptable. This constitutes a major advantage in stress related studies, opening the possibility of expressing genes of interest from the extremophile into *Arabidopsis* facilitating their characterization, and more importantly, their potential role in stress resilience.

The difference in genome size between Eutrema and *Arabidopsis* may be the key to the robust resilience mechanisms found in the extremophile, gene duplication mechanisms and transposable elements which increase genome size might be involved in evolutionary stress coping mechanisms [36], [49]. Yang et al. [41] compared gene copy number in TF families of stress-induced genes (SOS-like) between the two species and found that most of the TF families were present in approximately the same copy number. In fact, only the UBIQUITIN-DEPENDENT PROTEIN MODIFICATION gene family was shown to have a statistically significant increase in gene copy number in Eutrema. This evidence suggests that a highly stress-specific translational and transcriptional reprogramming occurs in Eutrema under abiotic stresses, which ultimately leads to metabolic and signalling changes [50]. According to Taji et al. [51], only six Eutrema genes were salt stress-inducible whose log_2_ ratios of salt vs. control were ≥1.5, whereas the same experiment in *Arabidopsis* showed 40 such salt stress-inducible genes. These differences are attributed to a constitutive high level of expression of stress-responsive genes in Eutrema in comparison with *Arabidopsis*. This constitutively high level of some transcripts is thought to contribute to its extreme salt tolerance. Another proposed hypothesis is that Eutrema has a photosystem that rapidly and efficiently responds to abiotic stress. Wong et al. [52] reported that genes exhibiting changes in transcription induced by cold stress are more similar between Eutrema and *Arabidopsis*, than either salt- or drought-induced sequences. Of the down regulated transcripts under cold stress 15% of them code for genes involved in photosynthesis, indicating that Eutrema has a significant capacity for mitigating the effects of photoinhibition [53].

### 4 Effects of cold stress in lipid remodelling

Lipid biosynthesis is composed of a multitude of processes that span different cellular organelles, such as plastids, the endoplasmic reticulum (ER), and cytosol, employing complex pathways and trafficking mechanisms. Fatty acid synthesis utilizes the carbon flux derived from photosynthesis in the form of pyruvate and it is localized in the plastid, after which the fatty acyl chains are then channelled to produce more complex lipid molecules in the plastid, or transported in the cytosol and endoplasmic reticulum (ER). This is regulated according to the supply and demand for acyl chains, and its production is balanced according to requirements. The regulation of fatty acid synthesis and further modifications is complex and has been extensively reviewed [54], [55].

The first line of physical defence against abiotic stresses is the leaf cuticle which incorporate intra- and epi-cuticular waxes. Cutin and suberin, present in plant cell walls, are complex fatty acid derived polymers that confer chemical and physical barriers that protect cells from external pathogens and control the movement of gases, water and solutes [56]. *Arabidopsis* sfr3 mutants which are sensitive to freezing, present an alteration of cuticle permeability due to the variation of wax composition and morphology, in the cold and in flower development. This phenotype arises from a missense mutation of the ACC1 gene, that encodes a cytoplasmic acetyl-CoA carboxylase (ACCase) involved in *de novo* fatty acid biosynthesis [57], [58].

Plasma membranes are considered as the primary barrier between the organism and external environment, being the first to experience the injurious effects of stress. Alterations in lipid composition/structure in the plasma membrane under environmental stresses are crucial to maintaining membrane stability and functions. In general, biological membranes have a crucial role not only in the protection, homeostasis and metabolism of the cell, but also in signal recognition and signalling cascades which are proposed as key regulatory processes both under optimum and stress conditions mediated by protein-lipid interactions. [8], [59].

### 4.1 Membrane remodelling

Cell membranes are composed of three main classes of lipids, glycerolipids (of which phosphoglycerolipids and galactoglycerolipids are the most abundance species in the extra plastidial membranes and chloroplast, respectively) sphingolipids and sterols. Whereas glycerolipids are synthesized on the cytosolic leaflet of the endoplasmic reticulum (ER), sphingolipid production is completed in the ER/Golgi, and later presented to the outer monolayer. With regard to sterols, they have a higher affinity for sphingolipids and are thought to fill the voids between the sphingolipids, increasing the packing of lipids [7]. Furthermore, the distribution of lipids within the plasma membrane is not uniform, rather sphingolipids and sterols segregate to form nanodomains, in which the packing of lipids is increased and a putative role in signal transduction processes and membrane trafficking has been assigned [7], [60].

Low temperature damage, also associated with freezing injury, can arise from changes in the intercellular water temperature which affects water potential through the plasma membrane, affecting also its own conformation causing structural damage and hence increasing ion leakage. Formation of non-bilayer lipid structures in both plasma and chloroplast membranes, such as the inverted hexagonal (HII)-type structures, can occur under cold temperatures causing lesions in the membrane. Under such conditions changes in the lipid composition, and membrane repair systems, mediated by many lipid pathway enzymes, are activated in order to maintain its structure, keeping membrane integrity and avoiding damage [8]. Plants often respond to low temperature stress by increasing membrane lipid unsaturation, chain shortening, altering lipid class composition and/or changing the lipid/protein ratio [9]. Certain molecular species are especially important and a good correlation has been noted for many plants where chilling sensitivity is associated with significant amounts of 16:0/16:0- or 16:0/*trans*-16:1-phosphatidylglycerol species [61]. Chilling tolerance in higher plants has been made possible by the introduction of desaturase genes [62], [63], [64], the desaturation of palmitate to palmitoleate or by modifying the biosynthetic pathway to phosphatidylglycerol where oleate (rather than palmitate) is acylated [65]. Apart from fatty acid unsaturation, phospholipid class composition can be affected e.g. sugar bean roots responded to low temperatures by lowering the ratio of phosphatidylcholine (PC) to phosphatidylethanolamine (PE). Changes in PC and PE after cold stress may be related to the activity of phospholipases. Phospholipase D (PLDα) can lead to a decline in PC rather than PE on sub-lethal freezing where an increase in phosphatidic acid (PA) and lysophospholipid is seen [66]. Phospholipase D (PLD) is activated by cold and increases the production of the signalling molecule PA [67], however, it is also proposed to have a mechanical role as a membrane anchor [68]. Belonging to the same family of enzymes, PLD from δ class (PLDδ) can bind microtubules and interact with the cytoskeleton stabilizing the membrane [69]. In *Arabidopsis*, pldδ mutants showed impaired freezing tolerance and reduced PA levels, whereas overexpression of this gene has the opposite effect [70]. The regulation of PLDδ and PLDα expression is driven by ACYL CoA-BINDING PROTEINS (ACBP6, ACBP1), which influence the PA signalling cascade and increase freezing tolerance [71], [72].

**Fig. 1:**
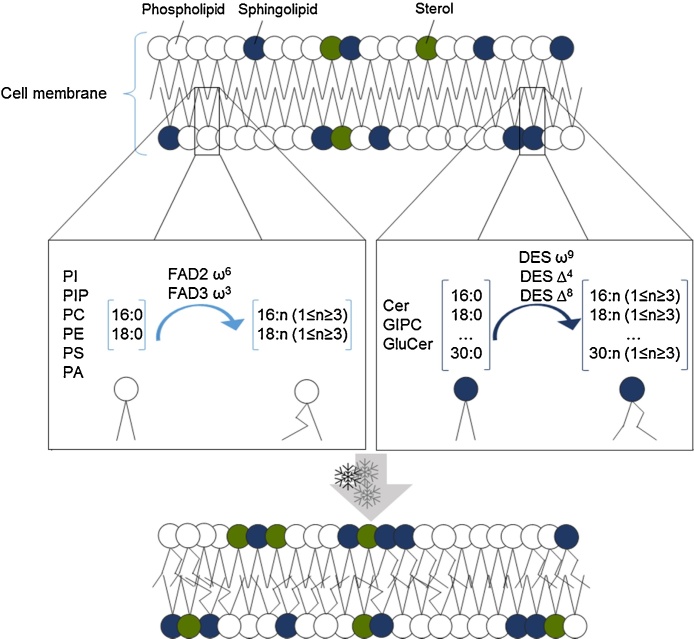
Schematic representation of the desaturation in phospholipids and sphingolipids species crucial for the remodelling of the plasma membrane under cold stress. PI – phosphatidylinositol; PIP – phosphoinositides; PC – phosphatidylcholine; PE – phosphatidylethanolamine; PS – phosphatidylserine; PA – phosphatidic acid; FAD – Fatty Acid Desaturase. Cer – Ceramides; GIPC – glycosylinositolphosphoryl ceramide; GluCer – glucosylceramide; DES – desaturase.

The ability to adjust membrane fluidity according to temperature changes is often attributed to the regulation of membrane fatty acid desaturation (Fig. 1) and chain length [10], [19], [73]. The increase in fatty acid unsaturation levels is mediated by fatty acid desaturases (FAD) located in the chloroplast and in the ER. There are up to seven FAD enzymes in plants with a significant role in the unsaturation level of membrane fatty acids, FAD2 and FAD3 effect extra-chloroplast lipid desaturation, whereas FAD4, FAD5, FAD6, FAD7 and FAD8 effect chloroplast desaturation [74]. By studying the fatty acid desaturase mutants in *Arabidopsis* (fad mutants) it was possible assign a role in cold tolerance to some of this genes [75]. When FAD2 ω6-oleate desaturase activity is supressed, *Arabidopsis* fad2 plants become chilling sensitive at 12 °C, and die when exposed to 6 °C [19]. In contrast, when FAD3 was overexpressed in *Arabidopsis*, transgenic plants were enriched in the linoleic acid content of the mitochondrial membranes, showing higher viscosity, and, hence, reduced rigidity with cold stress [76]. In addition, an increase of Δ8 unsaturation of the Long-Chain-Base (LCBs) of sphingolipids increased cold temperature tolerance in *Arabidopsis*. Although the specific mechanisms are yet to be understood, the role of this enzyme could be crucial in cold tolerance response and is a candidate worthy of further research [77]. Chain length has been shown to be regulated at least in part by ACC1. For example, the *Arabidopsis* sfr3 mutant is freezing sensitive due to a missense mutation in ACC1 (Acetyl-CoA carboxylase1). This enzyme is responsible for the cytosolic pool of malonyl-CoA to be used for the production of Very-Long-Chain-Fatty-Acid (VLCFA), essential for the assembly of complex lipids such as sphingolipids [57], [58].

The specific composition of the inner and outer chloroplast membranes also influences the cellular chilling response. The *Arabidopsis* COR15 proteins are encoded by two nuclear homologous genes COR15A and COR15B (82% sequence identity) and targeted to the chloroplast stroma [78], [79]. There they interact specifically with monogalactosyldiacylglycerol (MGDG) maintaining the structural orientation of the inner chloroplast envelope membrane lipids [80]. In *Arabidopsis* transgenic plants, overexpression of COR15 genes further stabilizes chloroplast membranes and increases freezing tolerance [81]. Chloroplast membrane stabilization is also maintained by the change in bilayer- to non-bilayer-forming membrane lipids carried out by SFR2. During freezing, SFR2 removes the galactose head group from MGDG and adds it to a second MGDG. This activity is processive, generating oligogalactolipids (digalactosyldiacylglycerol, trigalactosyldiacylglycerol, and up to hexagalactosyldiacylglycerol) and leaving diacylglycerol as a by-product. The diacylglycerol is converted into triacylglycerol (TAG); TAG and oligogalactolipids derived from MGDG specifically increase in response to freezing. The sfr2 *arabidopsis* mutants show extensive intracellular damage after freezing recovery, with rupture in both chloroplasts and tonoplasts likely through the fusion of destabilized membranes in these organelles [82], [83]. Research has further identified the chloroplast membrane as a site of cold perception, specifically the desaturation of membranes. The Acyl-lipid desaturases *1 and 2* genes (*ADS1 and ADS2*) genes are paralogous genes, sharing a 75% of similarity at the amino acid level, that encode cold inducible desaturases affecting cold acclimatization response and chilling/freezing tolerance, respectively [20], [84]. However, whereas *ADS1* encodes a soluble Δ9-desaturase that is localized exclusively in the chloroplast and catalyses the desaturation of stearic acid (18:1) of MDGD [84], *ADS2* encodes a 16:0 desaturase for MGDG and PG [84]. Both genes have been demonstrated to be essential for cold adaptation response in *Arabidopsis*, affecting chloroplast membrane desaturation.

### 4.2 Signalling for stress

The mechanical protection of biological membranes also has an important role in the production of stress responsive signalling lipids, which can be derived from membrane lipids, such as phospholipids and sphingolipids, through enzymatic reactions [22]. Signalling lipids represent less than 1% of total plant lipids characterized (by their transient accumulation and high turnover rate) and they have been shown to be derived from reactions involving phospholipases, lipid kinases or phosphatases, which produce mainly phosphatidic acid (PA), phosphoinositides (PtdIns), sphingolipids and free fatty acids (FFAs). Under freezing conditions a fast induction of PA occurs through the phosphorylation of PtdInsP to PtdIns(4,5)P2, which is then hydrolyzed by PLC into the second messengers Ins(1,4,5)P3, and DAG. The latter is then converted to PA by the action of DGK (diacylglycerol-kinases) [21], [85]. However, there is evidence that PtdIns(4,5)P2 may act as a stress signalling agent regulating cellular ion homeostasis [21], [86]. Furthermore, the PA induction in response to cold stress can also be derived by the hydrolysis of phospholipids, such as PC, PE, and phosphatidylglycerol (PG) [68]. The fast induction of the signalling molecules observed suggests a probable post-translational regulation of the enzymes involved in the process [67]. Although mainly thought to act as a signalling molecule recent discoveries have identified a functional role for PA as a second messenger molecule, PA has been shown to recruit target proteins with varied stress functions such as TFs, protein and lipid kinases, phosphatases and also cytoskeletal rearrangements [87], [88]. Furthermore, PA would also participate in the ABA cold response, through interaction with sphingosine kinases [89], that regulate the production of LCB-1-phosphates (LCBPs), including phyto-S1P, although the regulation mediated by this hormone is not fully understood.

Sphingolipids play an active role in cold transduction pathways. The fast and transient production of phyto-S1P, by a specific LCB kinase isoform (LCBK2), has been reported to have an effect in the regulation of the expression of cold-responsive gene expression [90], [91]. Furthermore, phosphorylated ceramides (Cer-P) have been found to be rapidly and transiently accumulate upon cold shock treatment. The ACCELERATED CELL DEATH5 (ACD5) gene, encodes a ceramide-kinase (Cer-K) responsible for spontaneous PCD that occurs in later stages of development in *Arabidopsis* knockout mutants (acd5) [92], [93]. These mutants have a 70% decrease in accumulation of Cer-P when compared to the wildtype, and they show hypersensitivity when germinated at low temperature [94]. The transient and rapidly accumulating characteristics reported for these molecules suggest a possible role as signal molecules, though the specific mechanisms and pathways are still to be investigated.

### 4.3 Transcriptional regulation of lipid related genes

Modulated responses to stress are regulated by stress-induced gene expression in a complex regulatory gene network mediated by TFs [95]. These can be activated via ABA-dependent or ABA-independent pathways, which can be distinguished by different recognition sites in the promoter of the induced gene. Although transcriptional regulation of the cold response has been studied in depth, only a small number of lipid-related TF genes has been described so far. Among others TT2 and TT8 (TRANSPARENT TESTA2 and 8), with corresponding TFs belonging to R2R3, MYB and bHLH families respectively, regulate proanthocyanidin and flavonoid synthesis in the seed coat [96]. Under cold stress TT2 and TT8 play opposing roles in lipid metabolism.TT2 negatively regulates FA accumulation in *Arabidopsis* seeds, and positively effects environmental stress tolerance during seed germination and plantlet establishment [97], whereas TT8 indirectly inhibits the expression of a series of genes in the FA biosynthetic pathway, including FATTY ACID BIOSYNTHESIS (FAB2), FATTY ACID ELONGATION1 (FAE1), FATTY ACID DESATURASES FAD2 and FAD3, important mediators of lipid-related cold responses [98]. The mechanism of upregulation of expression of these genes requires further investigation.

**Fig. 2:**
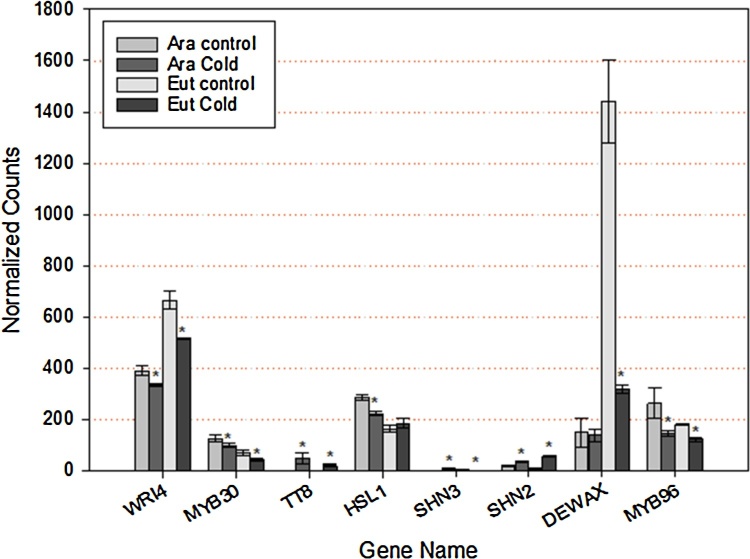
Expression levels of differentially expressed TFs involved in lipid metabolism (n = 4; ± standard error). Plants were grown at optimal conditions (control) at 23 °C day/18 °C night, 200 μmol m − 2 s − 1 light intensity, 16/8 light/dark photoperiod, and relative humidity 65%, were subjected to chilling (4 °C) for 3 days. Leaves of 22 days old plants were used total RNA extraction, and RNA-Seq analyses performed. TruSeq RNA libraries made and run of 4 lanes of 100 bp paired end Illumina Hi-Seq 2500 in Rapid run mode, generating 41–83 million reads per sample. This resulted in  ≈ 25000 genes with reads aligned in Arabidopsis (* represent differentially expression with a 5% significance level between control vs. cold).

To investigate the link between TF expression, lipids and chilling tolerance, we surveyed the Acyl Lipids database (aralip.plantbiology.msu.edu). To date 27 TFs related to lipid metabolism has been identified (Supplementary Table S1) [55]. To further understand their roles in configuring the low temperature response and identify additional TFs related to lipid metabolism with roles in the cold performance, we used transcriptomic results produced in our lab where *A. thaliana* Col-0 and *Eutrema salsuginea* where subject to 4 °C for 3 days (manuscript in preparation/unpublished data). We could identify reads for 17 of the 27 TFs, of which eight were differentially expressed in *Arabidopsis*, Eutrema or both. Those showing differences were predominantly associated with the fatty acid elongation and wax biosynthesis pathway (Fig. 2). Interestingly, most of the TFs analysed were downregulated when the plants were exposed to cold, with the exception of TT8, previously reported as implicated in lipid-related cold response [98]; and SH2 and SH3 which showed different responses. SH2 is induced in *Arabidopsis* and repressed in Eutrema, whilst SH3 is down-regulated in both species. Both TFs belong to the SHINE clade of AP2 domain TFs and activate wax biosynthesis, altering cuticle properties and conferring drought tolerance when overexpressed [99]. Thought to be functionally redundant when overexpressed, they differ in time and location of expression, which implies that they might play different roles both in normal conditions and under stress conditions [99]. Further investigation is required to explain the different behaviour of SH2 and SH3 in Eutrema when exposed to cold. Belonging to the same family of AP2 TFs, the expression of DEWAX gene is downregulated in Eutrema, whereas in *Arabidopsis* no changes were observed. The loss function mutant, *dewax*, leads to an increased wax load phenotype and it has been suggested that DEWAX-mediated transcriptional repression of wax biosynthesis genes [100]. The MYB TFs MYB30 and MYB96, which target genes are involved in wax biosynthesis, have been also show to be significantly down regulated in both species after cold treatment. Although not directly related to the cold stress response, both genes act by integrating hormone signalling, brassinosteroids and ABA/auxin for MYB 30 and MYB 96, respectively [101], [102]. These preliminary results suggest that altering the expression of the TFs involved in the regulation of WAX biosynthesis genes would be a good target for engineering increased cold stress resilience in Brassica species.

## 5 New challenges

Cold stress initiates a plethora of responses from the initial sensing and TF cascade to the cellular remodelling of membrane structures. Collectively these responses define the resilience of the plant to extremes of cold temperature. This review has sought to demonstrate the intimate role lipids play in defining the cells response to cold stress from protection to signalling. Indeed, lipids and their role in cold response mechanisms allow the plant to not only survive such stress, but to thrive under those conditions (e.g. Eutrema). Plant lipids are extensively studied mainly due to their economic significance as energy-storage compounds. More recently their active role on plant stress metabolism has been explored, although there remains still much to understand. The study of lipid biosynthetic pathways has benefitted greatly from improvements in mass spectrometry allowing the science of lipidomics to develop [103], [104]. By enabling the capture of the entire lipidome at any point in time from an individual tissue type, the resulting information can be used to compare species and treatments which are especially relevant to the study of abiotic stress.

Beyond the direct measurement of lipid species, Next-Generation Sequencing (NGS) has already revolutionized the study of transcriptomes [105]. and future work will likely utilize technologies such as genome editing (e.g. CRISPR/Cas9) to modify the genome in a precise manner [106], [107]. Such an approach will enable researchers to tease out the specific patterns and network clusters that enable the plant to negotiate extreme fluctuations in temperature. Furthermore, model species such as *Arabidopsis* have underpinned research into plant responses to perturbation, however, now much can be learned from the application of these approaches to extremophiles. However, to further establish *Eutrema salsugineum* as an extremophile plant model is still a challenge, and for the sake of reproducibility of experimental conditions and experiments, the improvement and adaption of existing protocols is essential. Techniques such as transformation [108], [109] need to be further adapted to the morphological and developmental characteristics of this species to achieve an optimized efficiency. Applying new techniques, such as the described CRISPR/Cas9 constitute both a challenge in the development and applicability of this method, and a defining strategy to make Eutrema a stress model plant.

Research into the response of the plant lipidome during periods of cold stress is a relatively recent area of work incorporating metabolite and transcriptomic approaches. The principle obstacle to overcome is the low level and high turnover of some (signalling) lipid species. Recent advances in the sensitivity and the accuracy of mass spectrometry and its linked chromatography are offering new insight into some of these hard to quantify lipids which when coupled with the power of RNA-Seq can identify the complex network of proteins that protect the plant from damage at low temperatures. The mechanism of action both in sensing low temperature and altering transcription and lipid turnover is unclear and further research must address the specific role of TFs in lipid homeostasis during cold stress. Addressing the question of whether the regulation of lipid species is predominantly transcriptional, translational or post-translational or a combination of these mechanisms will target the knowledge gap that presently exists and will enable the development of breeding strategies that deliver climate resilient crops.
